# Textile Retrieval Based on Image Content from CDC and Webcam Cameras in Indoor Environments

**DOI:** 10.3390/s18051329

**Published:** 2018-04-25

**Authors:** Oscar García-Olalla, Enrique Alegre, Laura Fernández-Robles, Eduardo Fidalgo, Surajit Saikia

**Affiliations:** 1 Department of Electrical, Systems and Automation, Universidad de León, 24071 León, Spain; ogaro@unileon.es (O.G.-O.); ealeg@unileon.es (E.A.); ssai@unileon.es (S.S.); 2 Department of Mechanical, Computer Science and Aerospace Engineering, Universidad de León, 24071 León, Spain; l.fernandez@unileon.es; 3 Researcher at INCIBE (Spanish National Cybersecurity Institute), 24005 León, Spain

**Keywords:** content-based image retrieval, textile retrieval, textile localization, texture retrieval, texture description, visual sensors

## Abstract

Textile based image retrieval for indoor environments can be used to retrieve images that contain the same textile, which may indicate that scenes are related. This makes up a useful approach for law enforcement agencies who want to find evidence based on matching between textiles. In this paper, we propose a novel pipeline that allows searching and retrieving textiles that appear in pictures of real scenes. Our approach is based on first obtaining regions containing textiles by using MSER on high pass filtered images of the RGB, HSV and Hue channels of the original photo. To describe the textile regions, we demonstrated that the combination of HOG and HCLOSIB is the best option for our proposal when using the correlation distance to match the query textile patch with the candidate regions. Furthermore, we introduce a new dataset, TextilTube, which comprises a total of 1913 textile regions labelled within 67 classes. We yielded 84.94% of success in the 40 nearest coincidences and 37.44% of precision taking into account just the first coincidence, which outperforms the current deep learning methods evaluated. Experimental results show that this pipeline can be used to set up an effective textile based image retrieval system in indoor environments.

## 1. Introduction

The process of automatically finding objects, textiles, faces, or other patterns in images and videos is one of the most studied topics in computer vision. Nowadays, with the huge amount of digital images and videos, it becomes even more critical. Visual sensors are able to acquire a large quantity of visual information from the surroundings around them. Content Based Image Retrieval (CBIR) consists of retrieving images using their content properties from a collection that match a user’s query [1] based on a similarity measure [2]. Many research fields, e.g., medical image [3,4,5], human retrieval [6], biological analysis [7,8], agricultural retrieval [9] and biometric security [10], achieved interesting results using CBIR techniques.

Most works related to CBIR aim at finding objects in datasets of images. Research groups face this problem using different approaches such as invariant local features (SIFT [11,12], SURF [13]), color description [14,15], template matching [16,17] or, more recently, deep learning techniques [18,19,20]. Nonetheless, these techniques may fail when the object does not present a rigid shape or it has a plain shape, as it is the case of textiles. The same textile can appear in images with very skewed shapes. Moreover, textile retrieval shares the difficulties of object retrieval such as the variety in illumination conditions, occlusions, lack of texture information, etc.

The need of retrieving textiles from image collections captured under a variety of visual sensors can be motivated by many applications [21]—for example, for marketing studies in textile stores that suggest the products that fit a decorated room to users. The recently published book “Applications of computer vision in fashion and textiles” [22] deals with three aspects related to computer vision techniques applied to textile industry: (i) textile defect detection and quality control, (ii) fashion recognition and 3D modeling, and (iii) 2D and 3D human body modeling for improving clothing fit. One of its chapters [23] reviews the computer vision state-of-the-art techniques for fashion textile modeling, recognition, and retrieval. A completely different approach in which textiles are needed to be retrieved is to connect evidence of different crime scenes.

In our case, this work is framed in the Advisory System Against Sexual Exploitation of Children (ASASEC) project, a European project that fights child pornography using forensic analysis, data mining and computer vision techniques. It was demonstrated that perverts usually use the same bedrooms to take their pictures or videos [24]. A way to link two images, and consequently provide relationships among the many cases of child pornography, is finding the exact same textiles such as carpets, blankets or any other repeated texture. In our specific case, we aim at evaluating textiles in order to retrieve images in huge datasets (thousands of images) of past proven cases of child abuse connected with a query textile of interest.

The rest of the paper is organized as follows. The related work is presented in Section 2. Section 3 describes the pipeline of our proposed method for textile based image retrieval. Section 4 introduces the TextilTube dataset, the evaluation metrics, the evaluation set-up and the decision evaluation of a distance measure. We show the results of all the experiments carried out in Section 5. Finally, Section 6 draws the conclusions of the paper.

## 2. Related Research

Material retrieval is related to textile retrieval in some aspects and it is more broadly studied in the literature. Zhu and Brilakis [25] presented a system for detecting concrete based on the account of the colour of the regions in the image. After that, they described the regions using color features and trained a machine learning classifier to determine if the region contains concrete or not. This method cannot deal with very heterogeneous regions due to the way of creating the image partitions. Son et al. [26] proposed a method based on ensemble classifiers in order to distinguish between concrete, steel and wood. One of the main disadvantages of this work is the necessity of uniform areas of the same material for the segmentation step, which consists of dividing the original image into sub-regions of a fixed size. If the material region is smaller than the grid division, a lot of information of the background is processed as a material resulting in a non-accurate description. In [27,28], the authors proposed two methods able to identify multiple materials in object surfaces without the need of segmentation. They first recognized the object class and then used correlations of material labels for such object. In this approach, the correct definition of detailed semantic cues of objects and materials is needed. In 2017, Xue et al. [29] focused on material recognition of real-world outdoor surfaces for which they presented a new very useful dataset for autonomous agents. They exploited the idea of extracting characteristics of materials encoded in the angular and spatial gradients of their appearance from images taken with small angular variations. We refer the reader to [30] for an overview of methods and applications for the automatic characterization of the visual appearance of materials. Material retrieval systems are effective when construction materials are involved, but they may fail with other kind of textiles. The main three differences with general textiles are: the classes of construction materials are well defined, the texture of the construction materials is more homogeneous and the image patches of construction materials are usually big and present regular shapes.

Besides material retrieval, there are also few textile retrieval works in the bulk of the literature. Bashar et al. [31] proposed a system based on three wavelet-domain based features called symmetry, regularity and directionality. In this paper, the authors demonstrated outperformance of the combination of the three features versus just the isolated descriptors using two datasets formed by 150 and 300 images of curtain patterns. Similarly, in 2009, Carbunaru et al. [32] proposed a method that applies independent component analysis over wavelet-domain images. In that case, the researchers chose a dataset composed of images of 30 different fabrics, obtaining an average recognition rate of 94.86%. Recently, in Chun et al. [33], a new method which uses composite feature vectors of color from spatial domain and texture from wavelet-transformed domain is proposed. In contrast with the other papers described before, Chun et al. carried out a retrieval system using a large dataset composed of 1343 textile images. In 2014, Huang and Lin [34] proposed a system based on the combination of color, texture and shape features in order to retrieve textiles over more than 4000 images downloaded from Globle-Tex Co., (http://www.globle-tex.com/). The retrieval system was based on a signature process extracted by different k-means clusters achieving an 83% of success rate. Nevertheless, in all cases, the material or textile datasets are already segmented, usually as a plain piece of fabric, and the system is only focused in the retrieval process. On the contrary, in our proposed work, the textiles are located in real environments presenting diverse shapes, under a wide range of capturing conditions and exposed to occlusions. Recently, a bunch of papers deal with query targets such as cloth worn on human bodies [35,36,37,38,39,40]. It is quite challenging to extract robust features from different images presenting different poses. Our paper encompasses a wider definition of the word textiles, and it is used to retrieve not only cloth on human bodies but any other textile that may appear in indoor environments.

The segmentation of regions of interest is thus critical for an efficient method. In 2016, Zheng and Sarem proposed a method called NAMES, which stands for Non-symmetry and Anti-packing Model and Extended Shading and is based on the idea of packing pixels with a very high performance in terms of time [41]. In 2015, Yang et al. [42] presented a method based on color histogram segmentation using HSV color space. In 2004, Matas et al. [43] proposed a method based on the extraction of Maximally Stable Extremal Regions (MSER) taking into account a binary threshold that varies along all the gray scale spectrum. In our work, we segmented our images using the latter method due to its tolerance against regions with little changes of intensity and the possibility of adjusting it using the binary threshold correctly. After the segmentation step, the description of the regions is another key step of our CBIR system. The detected textile regions can be described using texture descriptors, which are widely used for texture analysis. Texture analysis is a challenging and still open problem in computer vision that consists of detecting and describing the gray level spatial variations of the image pixels. Nowadays, there are multiple fields that profit from automatic texture retrieval, as it makes processes faster with no need for many qualified staff. For this purpose, local descriptors are yet extensively employed for texture description due to their high performance in terms of time and accuracy. Histogram of Oriented Gradients (HOG) is a very popular texture descriptor since Dalal and Triggs presented it in 2005 [44]. This method has demonstrated a great performance in multiple fields, such as pedestrian detection [44] or face recognition [45]. Another very popular descriptor is Local Binary Pattern (LBP) proposed by Ojala et al. [46] due to their simplicity and high capability to extract the intrinsic features from the textures. Guo et al. developed several modifications to LBP such as LBP variance (LBPV) [47], complete LBP (CLBP) [48] or adaptive LBP (ALBP) [49]. García-Olalla et al. introduced algorithms to enhance LBP description [50,51,52], developing a new booster method that can be fused with LBP in order to improve accuracy results [53]. We refer the reader to [54,55] for a general framework and a taxonomy of local binary patterns variants. Recent approaches are focusing on deep Convolutional Neural Networks (CNN) such as AlexNet [56], GoogleNet [57] or VGG-Net [58]. The activations generated at the fully connected layers are used as feature descriptors for image understanding [59], scene recognition [60], semantic segmentation [61], among others.

In this work, we propose a new method for textile based image retrieval in indoor scenes under diverse capturing conditions and subjected to different shapes and occlusions. In accordance with that goal, we present in this paper a new labeled dataset that we created and made publicly available (http://pitia.unileon.es/varp/node/483). It is composed of 684 images extracted from videos with 67 different classes of textiles and it is called TexilTube. We used videos recorded with different visual sensors, such as compact digital cameras (CDC) and webcams. This dataset reproduces, at a small scale, a typical scenario of image evidence related to child pornography. We used MSER on high pass filetered images of the RGB, HSV and Hue channels of the original images to extract the regions of textiles. To describe the textile regions, we used local texture features, i.e., LBP, ALBP, HOG and Faster R-CNN (Region based Convolutional Neural Network) [62]. To enhance the description of the texture descriptors, we used Complete Local Oriented Statistical Information Booster (CLOSIB) booster. We evaluated several distance measures, i.e., Spearman rank, Cityblock, Euclidean and Correlations distances and two evaluation metrics, i.e., precision at *n* and success at *n*. We consider the following contributions of this work: (i) we propose a method for extracting the regions of interest based on computing MSER on high pass filtered images of the RGB, HSV and Hue channels of the original images; (ii) we evaluated the performance several descriptors; (iii) we assessed several distance measures by means of a voting schema; and (iv) we present a new dataset for textile retrieval.

## 3. Method

### 3.1. Overview

**Figure 1:**
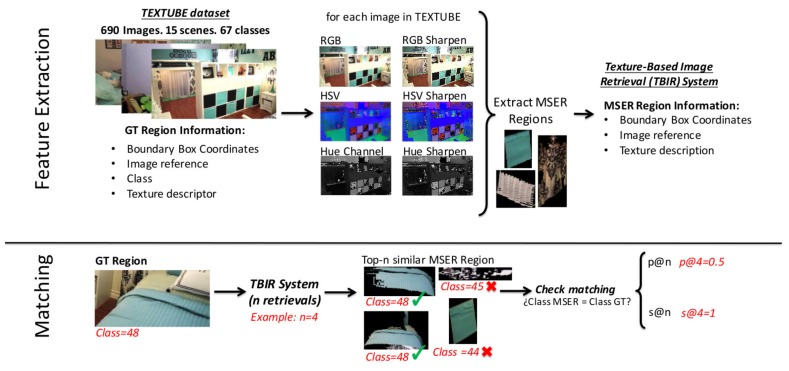
Scheme of the textile based image retrieval system.

In Figure 1, we illustrate the pipeline of our new method for textile based image retrieval. We can divide the method in two main stages: feature extraction and matching. By means of the experimentation carried out, we were able to determine that the regions in a scene containing textiles have to be extracted from three different transformations of the picture: a high pass filtered image of the RGB, HSV and Hue channels of the original image. We took this into consideration for building up the pipeline of the method.

The feature extraction is comprised of four steps. First, we convert the images to RGB and HSV colour spaces and we also extract the Hue channel. Then, we sharpen the image representations to increase the contrast along the edges where different colors meet. We adopt the unsharp masking method in which an image is sharpened by subtracting a blurred (unsharp) version of the image from itself. We use a Gaussian lowpass filter of standard deviation 1.5 for blurring the image. We use these three image representations for the extraction and description of the regions of interest in the images. The MSER [43] of the sharpened image representations define the regions of interest of the images. Finally, we describe the regions of interest by computing texture descriptors on the gray scale patch. We create a database in which we store for each detected region: the image coordinates of the bounding box of the region of interest in the image of reference, the images of reference themselves and the descriptors.

The matching stage allows for retrieving a given number of images that present the most similar regions to a query region (textile) of interest. It is made up of three steps. First, we describe the gray scale query region by means of the same texture descriptors. Second, we compute some distance measures among the descriptors of the query region and the descriptors of the database. Finally, the hit list is ranked by sorting the regions of the database in ascending order in relation with the distance measure.

Below, we briefly describe the methods used to build the novel pipeline.

### 3.2. Region Extraction: MSER

We use the MSER method [43] to automatically extract the regions (textiles) of interest due to the good results achieved in preliminary tests. Other methods apart from MSER could be evaluated for finding distinguished regions that possess some distinguishing or singular properties and allow for repeatedly detecting them over a range of image conditions, such as “sieve” [63]. However, an intensive evaluation of such methods is out of the scope of this paper.

MSER is a method for blob detection that extracts from an image a number of co-variant regions called MSERs. These high contrast regions are connected areas characterized by almost uniform intensity, surrounded by a contrasting background. MSERs are constructed by binarizing the image at multiple threshold levels and selecting the connected components that maintain their sizes over a large set of thresholds.

Experimentally, we chose to extract a great diversity of sizes for the areas of the regions of interest, specifically, comprehended between 3000 and 540,000 pixels, step size between intensity threshold levels equal to 3, and a maximum area variation between extremal regions of 0.7.

### 3.3. Region Description

We use the following methods to describe the textiles: LBP [64], ALBP [49] and HOG [44], and early fusion concatenations of the previous descriptors with CLOSIB and Half Complete Local Oriented Statistical Information Booster (HCLOSIB) enhancers [65].

#### 3.3.1. Local Binary Pattern (LBP)

LBP describes the texture of gray scale images by means of the local spatial structure on the image. For every pixel, a pattern code is computed by comparing its gray level value with the value of its neighbors.

In this work, we used uniform rotational invariant LBP [64] with 16 neighbors and a radius of two pixels, LBP${}_{16,2}^{riu2}$. The dimension of the descriptor is P+2, in this case, 16+2=18 elements. However, for simplicity, we call it LBP henceforth.

#### 3.3.2. Adaptive Local Binary Pattern (ALBP)

Guo et al. [49] presented a variation of LBP that considers the mean and the standard deviation along given orientation of the pixels in the image. This information is used in the matching step and makes it more robust against changes in the local spatial structure of the images.

We consider also the uniform rotation invariant version, ALBP${}_{16,2}^{riu2}$, and we call it ALBP for simplicity.

#### 3.3.3. Histogram of Oriented Gradients (HOG)

Histograms of Oriented Gradients [44] evaluates local histograms of image gradient orientations over a grid. HOG characterizes the local appearance of objects taking into account the local edge direction distributions. The method is implemented by dividing the image into small uniform regions called cells, often overlapped. Then, for each cell, a histogram of the gradient orientations over the pixels is extracted. The final descriptor is yielded by concatenation of the gradients along all the cells. In this work, we use overlapped cells of size 64×64 pixels on images resized to 256×256 pixels.

#### 3.3.4. Complete Local Oriented Statistical Information Booster (CLOSIB) Variants

CLOSIB [65] is obtained from the statistical information of the gray scale gradient magnitude of each pixel of the image. The statistical information of the gradient magnitudes is rarely taken into account to describe the image and provides useful information for texture classification. Equation (1) shows how to compute the CLOSIB enhancer of an image:

(1) $${\mathrm{CLOSIB}}_{P,R,\theta }=\overset{P/\eta }{\underset{p=1}{∥}}{\left(\left(\theta -1\right)\mu_{2}^{p}-{\left(-1\right)}^{\theta }{\left(\mu_{1}^{p}\right)}^{\theta }\right)}^{1/\theta },$$

where ∥ symbolizes the concatenation function, θ∈{1,2} is the order of the statistical moment considered, $\mu_{1}^{p}$ and $\mu_{2}^{p}$ are the first and second statistical raw moments, respectively, defined in Equation (2) and η is a factor that controls the portion of the considered orientations in the quantized angular space. We set η=1 for CLOSIB and η=2 for Half CLOSIB (HCLOSIB):

(2) $$\mu_{i}^{p}=\frac{1}{N}\sum_{c=1}^{N}{\left(|g_{c}-g_{p}|\right)}^{i},$$

where *N* is the number of pixels in the image, $g_{c}$ the gray value of the center pixel and $g_{p}$ the gray value of the neighbor, located at a distance *R* with orientation 2πp/P from the center pixel. In this work, we use the boosters $CLOSIB_{16,2,1}\left|\right|CLOSIB_{16,2,2}$ and $HCLOSIB_{16,2,1}\left|\right|HCLOSIB_{16,2,2}$, which we name CLOSIB and HCLOSIB, respectively, henceforth. Furthermore, we also use the early fusion (concatenation) of LBP, ALBP and HOG descriptors with CLOSIB and HCLOSIB enhancers to describe the texture of the textile regions. We denote the concatenation with the symbol +. For instance, LBP + CLOSIB stands for the early fusion of LBP and CLOSIB.

#### 3.3.5. Faster R-CNN

Faster R-CNN [62] is a Region based Convolutional Neural Network (R-CNN) that generates region of interest proposals by a Region Proposal Network (RPN). Faster R-CNN is basically composed of two parts: a RPN for creating a list of region proposals and a Fast R-CNN network [66] for classifying the regions into objects.

For the RPN, we applied a sliding window of size 3 × 3 on the features obtained at the last convolution layer, which yields an intermediate layer of dimension 512. We fed the intermediate layer into a box classification layer and a box regression layer. We fed the region proposals into a Fully Connected (FC) layer and we extracted the neural codes. Similarly to the MSER approach, we saved a database with the coordinates of the region proposals, the image reference and the neural codes. The matching step remains the same as for MSER approach. We used Faster R-CNN algorithm with VGG-16 [58] architecture pre-trained with an MS-COCO [67] dataset.

### 3.4. Distance Measures

We use five distance measures to compute the distances among the descriptors of the query region and the descriptors of the automatically detected regions of interest of the database. These are: Spearman, Cosine, Cityblock, Euclidean and Correlation distances. Spearman rank correlation coefficient is a nonparametric measure of rank correlation and it measures the strength and direction of association between two ranked variables. This measure uses a variable’s rank which is the average of their positions in the ascending order of the values. Spearman rank correlation coefficient of two vectors *A* and *B* is mathematically defined in Equation (3):

(3) $$d_{spe}\left(A,B\right)=1-\frac{(\left(r\left(A\right)-\bar{r(A)}\right){\left(r\left(B\right)-\bar{r(B)}\right)}^{T}}{\sqrt{(\left(r\left(A\right)-\bar{r(A)}\right){\left(r\left(A\right)-\bar{r(A)}\right)}^{T}}\sqrt{(\left(r\left(B\right)-\bar{r(B)}\right){\left(r\left(B\right)-\bar{r(B)}\right)}^{T}}},$$

where $\bar{r(A)}$ and $\bar{r(B)}$ are the mean value of the ranked vector *A*, r(A), and ranked vector *B*, r(B), respectively. The superscript *T* indicates the transpose of the matrix. Hereafter, we keep the same notation. Cosine distance calculates the angular cosine between two vectors following Equation (4):

(4) $$d_{cos}\left(A,B\right)=1-\frac{AB^{T}}{\sqrt{\left(AA^{T}\right)\left(BB^{T}\right)}}.$$

Cityblock distance is calculated using Equation (5) and is defined by the sum of the absolute distances of every coordinate between two vectors. *n* is the dimension of the vectors. This measure distance depends on the rotation of the coordinate system but is invariant to reflection and translation:

(5) $$d_{cit}\left(A,B\right)=\sum_{j=1}^{n}\left|A_{j}-B_{j}\right|.$$

Euclidean distance (see Equation (6)) is the most commonly used distance measure and calculates the length of the straight segment that connects two vectors:

(6) $$d_{euc}\left(A,B\right)=\sqrt{(\left(A-B\right){\left(A-B\right)}^{T}}.$$

The Correlation distance is obtaining by dividing the distance covariance of two vectors by the product of their distance standard deviations. See Equation (7):

(7) $$d_{cor}\left(A,B\right)=1-\frac{\left(A-\bar{A}\right){\left(B-\bar{B}\right)}^{T}}{\sqrt{\left(A-\bar{A}\right){\left(A-\bar{A}\right)}^{T}}\sqrt{\left(B-\bar{B}\right){\left(B-\bar{B}\right)}^{T}}}.$$

## 4. Experiments

### 4.1. TextilTube Dataset

Textile retrieval in real environments is a poorly investigated research field besides fashion cloth retrieval. Up to our knowledge, there is no publicly available dataset that focuses on the recognition of rigid and non-rigid textiles presented in different sizes, shapes and capturing conditions. For this reason, we created a new dataset for the retrieval of textiles in bedrooms (http://pitia.unileon.es/varp/node/483).

**Figure 2:**
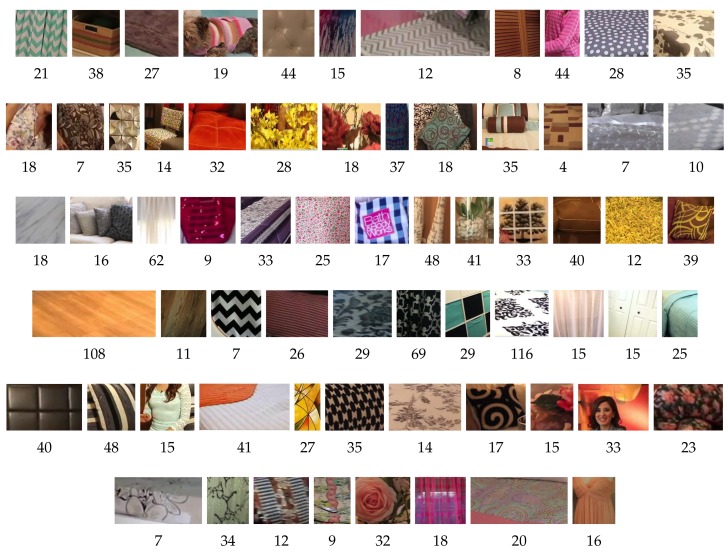
A region sample of each of the 67 classes in TexilTube dataset. The number underneath indicates the amount of regions that belong to that class.

The dataset is composed of 684 images of sizes that range between 480 × 360 and 1280 × 720 pixels obtained from 15 videos of YouTube. The videos were recorded in bedrooms with different visual sensors, such as CDCs and webcams. The videos contain plenty of textiles, different camera poses, illumination conditions, occlusions, etc., which makes the textile retrieval task very challenging. The dataset contains 67 classes of textiles such as curtains, carpets, sofas, shirts or dresses, among others. In one image, several classes of textiles may appear. Figure 2 shows a mosaic encompassing one region sample of each class and indicates the number of regions in each class. The number of elements of each class varies from 4 to 116. There is a total of 1913 regions. Therefore, the dataset is highly skewed, simulating a real scenario.

**Figure 3:**
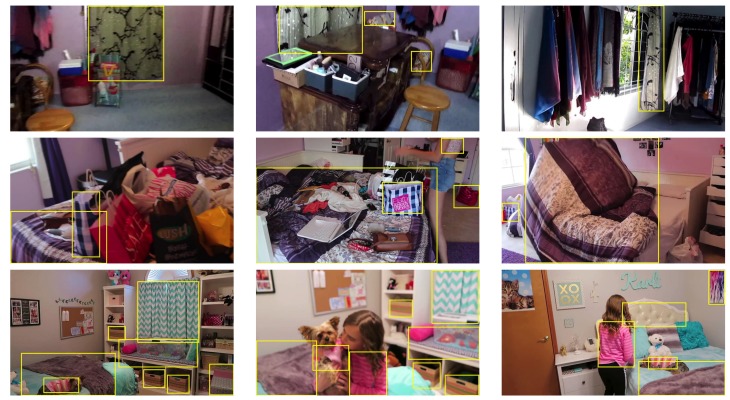
In rows, images that contain the same textile class in TextilTube dataset. The yellow rectangles overlaid in the images indicate the bounding boxes of the textile regions of the ground truth.

We labelled the dataset in order to provide a ground truth that allows the user to automatically evaluate the performance of a method on the dataset. The ground truth includes the bounding box coordinates and the class labels of each textile region in the images of the dataset. We provide the ground truth in the form of an XML file. We show the diversity in terms of type, size, pose, etc. of some textile classes of the dataset in Figure 3.

TextilTube dataset can be very interesting in fields like child sexual abuse or robbery to connect evidence of different investigations and also for marketing studies in textile stores to suggest the products that best fit the decoration of users’ rooms.

### 4.2. Performance Evaluation Metrics

In retrieval systems, it is important that the retrieved images are ranked according to their relevance to the query region forming a hit list, rather than being returned as a set. The most relevant hits must be within the top images of the hit list returned for a query region. To account for the quality of ranking the hits in the hit list, we used relevance ranking measures, i.e., precision at *n* and success at *n*.

#### 4.2.1. Precision at *n*

Precision at *n*, *p*@*n*, is the rate of the top-*n* images of the hit list correctly classified in relation to the class of the query region. Likewise, the precision at a cut-off of *n* elements of the hit list. We define $HitList_{n}$ as the set that contains the *n* images with smallest distance to the query region, *q*. Equation (8) presents the mathematical definition of precision at *n*:

(8) $$p@n=\frac{\#H(q)}{n},$$

where #H(q) is the cardinal of $HitList_{n}$ in which the query class is actually present in the image and the detected region overlaps the bounding box of the ground truth. It is formally defined in Equation (9):

(9) $$H\left(q\right)=\left\{h_{i}/\left(h_{i}\in HitList_{n}\right)\wedge \left(class\left(h_{i}\right)=class\left(q\right)\right)|i=1\ldots n\right\},$$

where $h_{i}$ is *i*-th retrieved image in the hit list.

#### 4.2.2. Success at *n*

There are occasions in which the user does not need to see many relevant images but is disappointed by a completely irrelevant top-*n* [68]. This is the case of the ASASEC project, in which finding at least one hit in all the hit list would be a satisfactory result. Success at *n*, *s*@*n*, measures if a relevant image was retrieved within the top-*n* hits of the hit list. Success at *n* is equal to 1 if the top-*n* images contain a relevant document and 0 otherwise (see Equation (10)):

(10) $$s@n=\begin{array}{ll} 1, & \mathrm{if}H(q)\neq \emptyset ,\\ 0, & \mathrm{otherwise}, \end{array}$$

where H(q) is the set of images defined in Equation (9).

### 4.3. Experimental Setup

We applied the method described in Section 3 to the 684 images of TextilTube dataset, extracting a total of 58,031 regions. In order to evaluate the performance of our method, we used the ground truth textile regions as query regions of interest. For each query region, we calculated *p*@*n* and *s*@*n* metrics for the retrieved hit list when computing a given distance measure among the texture descriptors of the query region and the analogous texture descriptors of the database. Experiments using Faster R-CNN were developed using the Caffe [69] deep learning framework in Nvidia Titan X GPU https://www.nvidia.com/en-us/geforce/products/10series/titan-x-pascal/.

### 4.4. Distance Measure Evaluation

**Figure 4:**
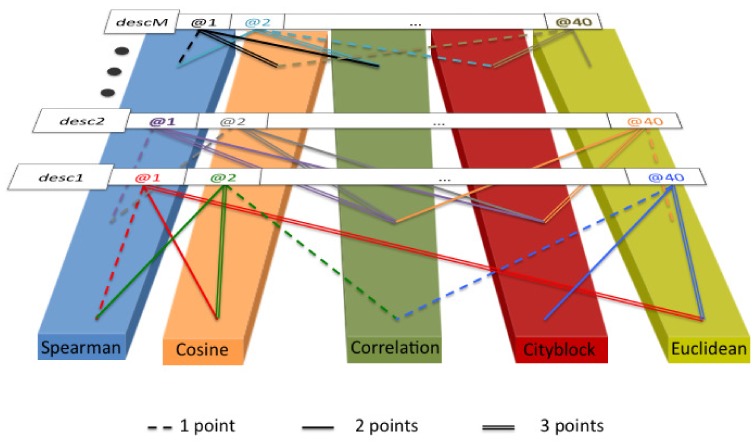
Scheme of the voting procedure to determine the best distance measure.

In order to determine the best distance measure and present uniform results, we carried out the following voting system. For each texture descriptor in Section 3.2, we computed *s*@*n* for $n\in N|n=\left\{1,2,\cdots ,40\right\}$ with all distance measures described in Section 3.4. We assigned three, two and one points to the distance measures that achieved the highest, second highest and third highest *s*@*n*, respectively, for each experiment. Finally, we summed up the points along all combinations. Figure 4 shows a scheme of the procedure. We disregarded a voting system that only relies on the best distance measure of each experiment because the results for the different distance measures were not enough distinctive.

**Figure 5:**
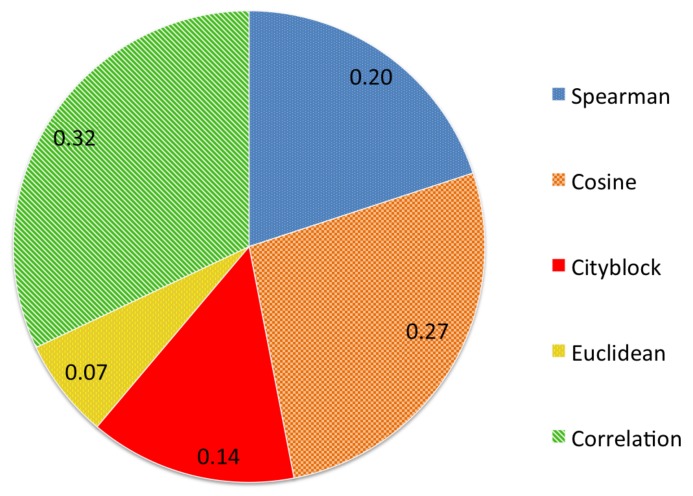
Results of the voting process in parts per unity for the different distance measures.

Figure 5 presents the results in parts per unity achieved with each distance measure. Correlation distance achieved the best results with a 32% of votes, followed by Cosine distance (27%) and Spearman rank correlation coefficient (20%). The commonly used Euclidean distance only yielded a 7% of the votes. Therefore, we carried out our experiments using the Correlation distance.

## 5. Results

In this section, we present the results obtained following the proposed method and experimentation for each evaluated texture descriptor and the neural codes extracted by Faster R-CNN.

**Figure 6:**
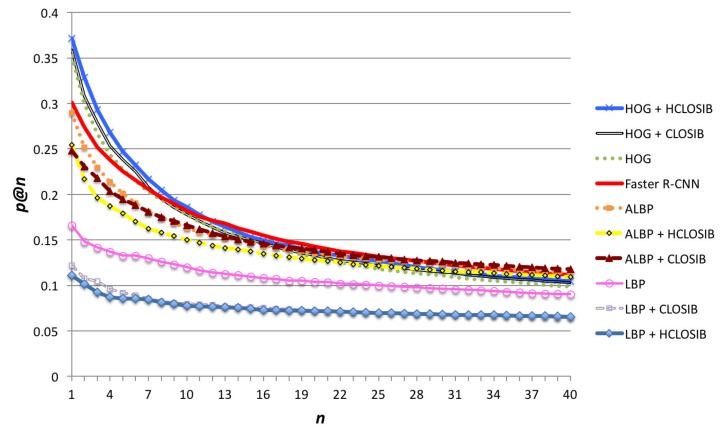
Precision at *n* (*p*@*n*) for all texture descriptors using Correlation distance and $n\in N|n=\left\{1,2,\cdots ,40\right\}$.

Figure 6 shows the precision at *n* (*p*@*n*) achieved for all texture descriptors. We used values of $n\in N|n=\left\{1,2,\cdots ,40\right\}$. For n≤11, HOG + HCLOSIB descriptor outperformed the rest with a precision of 37.17% for n=1. The early fusion of CLOSIB and HCLOSIB with HOG outperforms HOG alone. However, the early fusion of CLOSIB and HCLOSIB with LBP obtained the worst results. In the case of ALBP, the descriptor alone outperforms the early fusion for small values of *n*, whereas the opposite is true for high values of *n*. It is worth noting the better performance of ALBP (28.83% for n=1) versus LBP (16.60% for n=1). At a cut of 20, the precision at *n* values starts to stabilize. We present the numerical results for precision at cuts $\left\{1,2,\cdots ,20\right\}$ in Table 1. For high cuts of the hit list, Faster-RCNN slightly outperformed the rest. The best performance was not achieved by some LBP variant as we expected, but by HOG combined with HCLOSIB. HOG is oriented to gather the external and internal shape and HCLOSIB represents the statistical distributions of the texture. The combination of both represents both the shape of the textile’s texture (HOG) and how this texture is organized along the evaluated patch.

**Figure 7:**
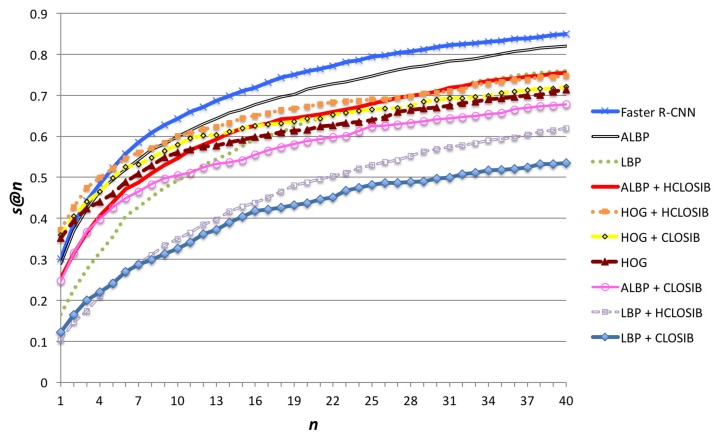
Success at *n* (*s*@*n*) for all texture descriptors using Correlation distance and $n\in N|n=\left\{1,2,\cdots ,40\right\}$.

Figure 7 illustrates the success at *n* (*s*@*n*). As expected, *s*@1 is the same as *p*@1 and for higher cuts of the hit list the success metric increases. In Table 2, we show the numerical results for success at cuts $\left\{1,2,\cdots ,20\right\}$. For values of n≤5, HOG + HCLOSIB yielded the best results, whereas for higher values of *n*, Faster R-CNN outperformed the others with a 84.94% of s@40 (74.86% with HOG + HCLOSIB). ALBP is the second best descriptor for n=40 reaching 82.00% of success. CLOSIB enhancer improves the performance of HOG and decreases the performance of LBP and ALBP.

**Figure 8:**
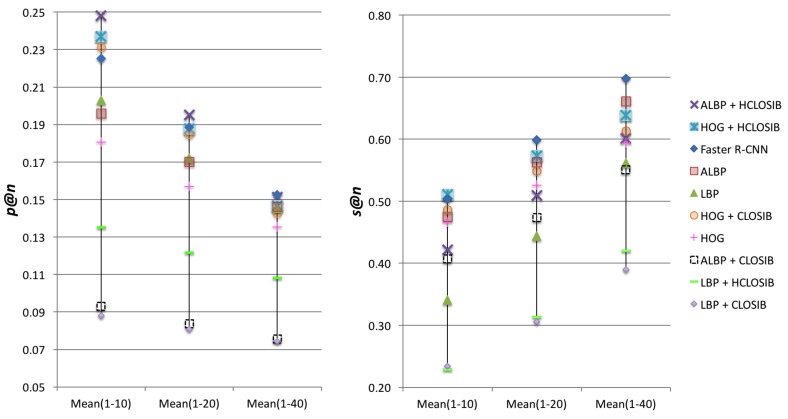
Arithmetic mean of precision and success at *n* for intervals of *n* from 1 to 10, from 1 to 20 and from 1 to 40.

In order to get a unique value to evaluate the performance of each descriptor, we computed the arithmetic mean of the success and precision for three intervals of $n\in N|n=\left\{1,2,\cdots ,j\right\}$, where $j=\left\{10,20,40\right\}$. Figure 8 and Table 3 show the arithmetic mean of the success and the precision in these intervals of values of *n*. Regarding *p*@*n*, HOG + HCLOSIB outperformed the rest of descriptors for the intervals with j=10 and j=20, whereas Faster-RCNN obtained the best results for j=40. With respect to *s*@*n*, HOG + HCLOSIB yielded the best results for the intervals with j=10, whereas Faster R-CNN did for j=20 and j=40. In such a difficult dataset, the outlined method using HOG + HCLOSIB descriptor and Correlation distance measure yielded an arithmetic mean of the precision at 10 of 24.80% and an arithmetic mean of the success at 10 of 51.08%.

**Figure 9:**
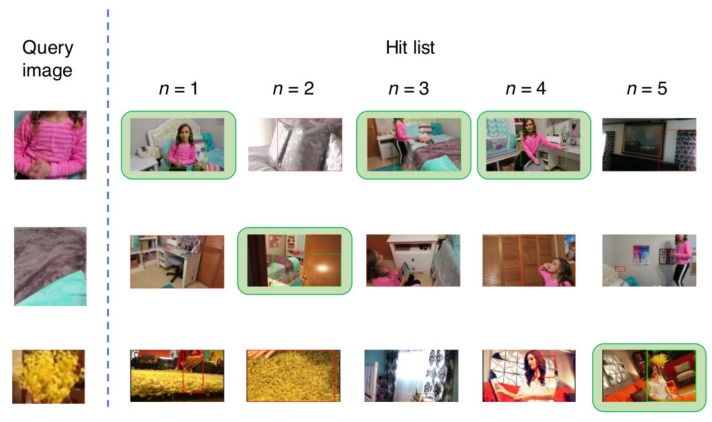
First five retrieved images in the hit list for three query samples using HOG + HCLOSIB.

**Figure 10:**
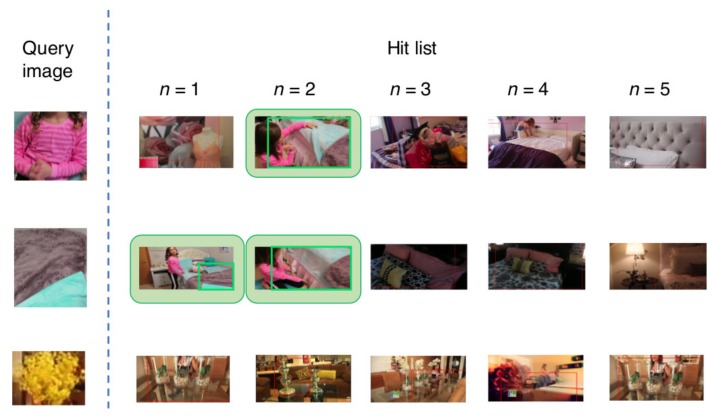
First five retrieved images in the hit list for three query samples using Faster R-CNN.

Figure 9 and Figure 10 show the visual results for the first five retrieved images in the hit list using HOG + HCLOSIB and Faster R-CNN, respectively. The third textile, artificial flower textile, is a difficult case. HOG + HCLOSIB manages to get one correctly retrieved images for n=5. The first two retrieved images are similar textiles of a rug. For the same query image, Faster R-CNN does not retrieve any correct images at a cut of hit list of n=5. Pre-trained deep neural networks are trained to classify objects instead of textiles. When a textile does not present a patterned texture, a pre-trained Faster R-CNN is not appropriate to retrieve such queries. However, Faster R-CNN manages to retrieve textiles correctly that present distinctive patterns.

## 6. Conclusions

In this paper, we presented a new application for textile based image retrieval in indoor environments. Textile based image retrieval is barely studied and when doing so, it is usually applied to fashion cloth retrieval. We introduced a new framework of study, the fight of child sexual abuse. Law enforcement agencies are interested in relating evidence by using textile queries in order to retrieve images or videos that contain the same textile in proven cases of child pornography, usually taken from rooms of houses. We proposed a new effective method for textile based image retrieval in rooms based on texture description of the MSER regions of the images. We assessed LBP, ALBP, HOG and their combination with CLOSIB for describing the image patches and several distance metrics for sorting the hit list. We also evaluated the Faster R-CNN algorithm with VGG-16 architecture pre-trained with MS-COCO dataset. Furthermore, we created and introduced a new public dataset, TextilTube, which consists of 684 frames from 15 Youtube videos of rooms recorded with different visual sensors. The dataset contains 1913 regions of interest that highly vary in terms of capturing conditions, occlusions, illuminations, etc. Moreover, textiles appearing in the images are not rigid and present different shapes. Correlation distance proved to be the most discriminant distance measure based on a voting system analysis. Correlation distance achieved 32% of the votes followed by cosine distance with 27%. HOG + HCLOSIB yielded the best results for low cuts of the hit list, whereas Faster R-CNN performed better for high cuts, closely followed by ALBPS. Taking into account just the most similar image retrieved, HOG + HCLOSIB achieved a precision of 37.17%, which is remarkable due to the number of classes in the dataset (67 classes) and their high intra-class variability. Taking into account the success at *n* metric, Faster R-CNN achieved a 84.94% retrieving 40 images (ALBP obtained a 82%), which means that about 85 out of 100 images have at least one correspondence in the top 40 retrieved images. This is a very interesting result that can be presented as an application for the criminal police in order to let them evaluate a grid of 40 images at a glance to check if there is a real match to the query image within the hit list. For the application at hand, it is interesting to achieve a high precision at low cuts of the hit list in order to reduce the number of images to visually inspect. To measure this fact, we computed the arithmetic mean of precision at *n* from 1 to 10. HOG + HCLOSIB outperformed the rest yielding a 24.8% hit rate. The main problem in this application is to find regions containing textiles, rather than objects. To the best of our knowledge, all the deep learning region proposal models are oriented and trained to detect objects. Objects are usually non-homogeneous regions as opposed to textiles. The reason is that CBIR systems are oriented to retrieve objects but not textiles or similar surfaces. Similarly, the deep learning models, are trained with datasets such as MS-COCO or ImageNet, among others, that contain objects and different classes of objects and they are oriented for instance retrieval. In future works, we will train a model for proposing regions with a textile dataset to strengthen the use of deep learning for textile retrieval. Besides evaluating other Region Proposal Networks in future works, different alternatives to MSER for finding distinguished regions, such as Sieve, will be also tested.

## Figures and Tables

**Table 1:** Precision at *n* (*p*@*n*) for all texture descriptors using Correlation distance and $n\in N|n=\left\{1,2,\cdots ,20\right\}$. Results highlighted in bold mark the best results per cut of the hit list.

Descriptor	*n*									
	1	2	3	4	5	6	7	8	9	10
HOG + HCLOSIB	**37.2**	**32.8**	**29.3**	**26.8**	**24.7**	**23.2**	**21.7**	**20.5**	**19.4**	**18.6**
HOG + CLOSIB	35.9	30.8	27.9	25.4	23.8	22.5	20.8	19.5	18.5	17.7
HOG	35.2	30.0	26.7	24.4	22.8	21.5	20.2	19.4	18.7	17.8
Faster R-CNN	30.1	27.4	25.2	23.8	22.5	21.6	20.6	19.7	19.0	18.2
ALBP	28.9	25.2	23.0	21.4	20.1	19.0	18.1	17.5	16.9	16.3
ALBP + HCLOSIB	25.5	21.7	19.6	18.7	17.9	17.0	16.2	15.8	15.4	15.0
ALBP + CLOSIB	24.8	23.1	21.8	20.4	19.5	18.8	18.1	17.5	17.1	16.6
LBP	16.6	14.8	14.2	13.7	13.3	13.3	13.0	12.6	12.3	12.0
LBP + CLOSIB	12.2	10.8	10.4	9.6	9.2	8.9	8.5	8.3	8.1	7.9
LBP + HCLOSIB	11.1	10.1	9.3	8.7	8.6	8.5	8.4	8.1	8.0	7.7
**Descriptor**	***n***									
	**11**	**12**	**13**	**14**	**15**	**16**	**17**	**18**	**19**	**20**
HOG + HCLOSIB	**17.7**	17.0	16.4	15.8	15.3	15.0	14.6	14.4	14.0	13.7
HOG + CLOSIB	17.1	16.4	15.8	15.4	14.9	14.6	14.2	13.9	13.6	13.3
HOG	17.0	16.4	15.9	15.5	14.9	14.5	14.1	13.7	13.4	13.0
Faster R-CNN	17.6	**17.1**	**16.8**	**16.3**	**15.9**	**15.5**	**15.1**	**14.8**	**14.6**	**14.3**
ALBP	15.9	15.6	15.2	15.0	14.7	14.4	14.2	14.0	13.8	13.5
ALBP + HCLOSIB	14.7	14.4	14.1	14.0	13.7	13.5	13.3	13.1	13.0	12.8
ALBP + CLOSIB	16.2	15.7	15.4	15.1	14.8	14.6	14.4	14.2	14.0	13.9
LBP	11.7	11.4	11.3	11.1	11.0	10.8	10.7	10.5	10.5	10.4
LBP + CLOSIB	7.9	7.8	7.7	7.6	7.6	7.5	7.4	7.4	7.3	7.2
LBP + HCLOSIB	7.7	7.7	7.6	7.5	7.5	7.3	7.3	7.2	7.2	7.2

**Table 2:** Success at *n* (*p*@*n*) for all texture descriptors using Correlation distance and $n\in N|n=\left\{1,2,\cdots ,20\right\}$. Results highlighted in bold mark the best results per cut of the hit list.

Descriptor	*n*									
	1	2	3	4	5	6	7	8	9	10
Faster R-CNN	30.1	38.7	44.3	48.4	**52.2**	**55.7**	**58.6**	**60.9**	**62.8**	**64.3**
ALBP	28.9	37.0	42.3	46.7	49.5	51.9	54.4	56.8	58.2	60.0
LBP	16.6	22.6	27.6	31.7	35.5	40.5	42.8	45.2	47.5	49.2
ALBP + HCLOSIB	25.5	31.4	36.5	40.6	44.0	47.1	48.8	51.0	53.0	54.7
HOG + HCLOSIB	**37.2**	**42.8**	**47.5**	**50.0**	**52.2**	54.6	56.1	57.1	58.4	60.1
HOG + CLOSIB	35.9	40.6	44.0	46.6	49.8	52.5	53.2	54.8	56.4	57.9
HOG	35.2	39.5	42.4	44.1	46.1	48.9	50.9	52.9	54.9	56.1
ALBP + CLOSIB	24.8	31.7	36.7	39.8	42.7	49.0	46.6	48.3	49.8	50.5
LBP + HCLOSIB	10.4	14.7	17.4	21.1	24.4	27.1	29.0	31.1	33.4	34.9
LBP + CLOSIB	12.2	16.5	20.0	22.0	24.1	26.9	28.7	29.9	31.3	32.6
**Descriptor**	***n***									
	**11**	**12**	**13**	**14**	**15**	**16**	**17**	**18**	**19**	**20**
Faster R-CNN	**65.9**	**67.2**	**68.7**	**69.9**	**71.1**	**71.9**	**73.1**	**74.3**	**75.0**	**75.9**
ALBP	61.5	63.0	64.3	65.8	66.5	67.8	68.7	69.6	70.2	71.5
LBP	50.9	53.0	54.5	55.8	57.6	59.4	60.2	61.0	62.4	63.5
ALBP + HCLOSIB	56.6	57.9	59.2	60.8	61.9	62.6	63.1	64.2	64.5	64.9
HOG + HCLOSIB	60.8	61.7	62.3	63.3	64.5	65.0	65.6	66.5	66.8	67.3
HOG + CLOSIB	59.5	60.2	60.4	61.2	62.0	62.6	62.9	63.2	63.7	64.3
HOG	56.8	57.5	57.7	58.6	59.1	59.9	60.3	61.0	61.4	61.6
ALBP + CLOSIB	51.2	52.5	53.3	53.7	54.2	55.6	56.6	57.4	58.1	58.8
LBP + HCLOSIB	36.5	38.5	39.7	41.7	42.9	43.9	45.1	46.6	48.1	48.7
LBP + CLOSIB	34.2	36.1	37.2	38.9	40.4	41.8	42.1	42.7	43.2	43.7

**Table 3:** Arithmetic mean of precision and success at *n* for intervals of *n* from 1 to 10, from 1 to 20 and from 1 to 40. Results highlighted in bold mark the best results per performance metric.

Descriptor	Precision			Success		
	Mean (1–10)	Mean (1–20)	Mean (1–40)	Mean (1–10)	Mean (1–20)	Mean (1–40)
HOG + HCLOSIB	**24.8**	**19.5**	15.1	**51.1**	57.3	63.9
HOG + CLOSIB	23.7	18.8	14.7	48.7	54.9	61.4
HOG	23.1	18.5	14.3	46.6	52.6	59.4
Faster R-CNN	22.5	18.8	**15.3**	50.3	**59.9**	**69.8**
ALBP	20.3	17.2	14.5	47.5	56.4	66.1
ALBP + HCLOSIB	18.1	15.7	13.5	42.2	50.9	60.1
ALBP + CLOSIB	19.6	17.0	14.6	40.7	47.3	55.1
LBP	13.5	12.2	10.8	34.1	44.4	56.1
LBP + CLOSIB	9.3	8.4	7.5	23.4	30.6	39.0
LBP + HCLOSIB	8.8	8.1	7.4	22.8	31.3	42.0

## Abbreviations

The following abbreviations are used in this manuscript:

LBP	Local Binary Pattern
ALBP	Adaptive Local Binary Pattern
HOG	Histogram of Oriented Gradients
CLOSIB	Complete Local Oriented Statistical Information Booster
HCLOSIB	Half Complete Local Oriented Statistical Information Booster
MSER	Maximally Stable Extremal Regions
CBIR	Content Based Image Retrieval
ASASEC	Advisory System Against Sexual Exploitation of Children
HSV	Hue, Saturation, Value
CDC	Compact Digital Cameras
*p*@*n*	precision at *n*
*s*@*n*	success at *n*
CNN	Convolutional Neural Networks
R-CNN	Region based Convolutional Neural Network
RPN	Region Proposal Network
FC	Fully Connected
RGB	Red, Green, Blue
SIFT	Scale-Invariant Feature Transform
SURF	Speeded Up Robust Features
VGG	Visual Geometry Group
MS-COCO	MicroSoft Common Objects in COntext
XML	eXtensible Markup Language
